# The utility of 3D printed models in complex percutaneous paravalvular leak interventions

**DOI:** 10.21542/gcsp.2020.27

**Published:** 2020-11-30

**Authors:** Ahmed ElGuindy, Ahmed Osman, Ahmed Elborae, Mohamed Nagy

**Affiliations:** 1 Aswan Heart Centre, Aswan, Egypt; 2 Faculty of Medicine, Cairo University, Egypt

## Abstract

Paravalvular leaks (PVL) are seen in 5–17% of patients after surgical mitral and aortic valve replacement. This is usually well-tolerated in the majority of patients; however, up to 5% will require re-intervention due to either hemodynamically significant regurgitation or hemolysis requiring repeated blood transfusion. Transcatheter closure of PVLs is becoming the treatment of choice in many patients owing to the high risk of redo surgery, high rates of recurrence with the surgical approach, and substantial improvements in device technology and growing expertise in structural heart disease interventions. Careful selection of the appropriate candidates by the Heart Team with in-depth analysis of clinical and multimodality imaging data is critical to ensuring good short- and long-term outcomes^1^.

The defect is usually oval/crescentic and often serpiginous in nature, which poses significant challenges in choosing the optimal size and number of devices to implant - especially with large size defects. Generally, defects involving more than 25–30% of the sewing ring are deemed unsuitable for percutaneous closure. While the Amplatzer family of vascular plugs (e.g. AVP3 and AVP2) is commonly used for percutaneous closure of PVLs, there are currently no approved dedicated devices for this indication, except the paravalvular leak device (Occlutech) which is not universally available. Small and relatively circular defects can usually be closed using a single plug, conventionally utilizing a size that is 25–30% larger than the mean diameter of the defect. Larger and crescentic defects on the other hand frequently require more than one plug and can be quite challenging in terms of choosing the appropriate size(s)^2^.

We report two cases with very large defects with irregular shape in which 3D printed modeling was extremely useful for bench testing to optimize the number and sizes of devices to be implanted.

## Case One

### Clinical vignette

**Figure 1.**
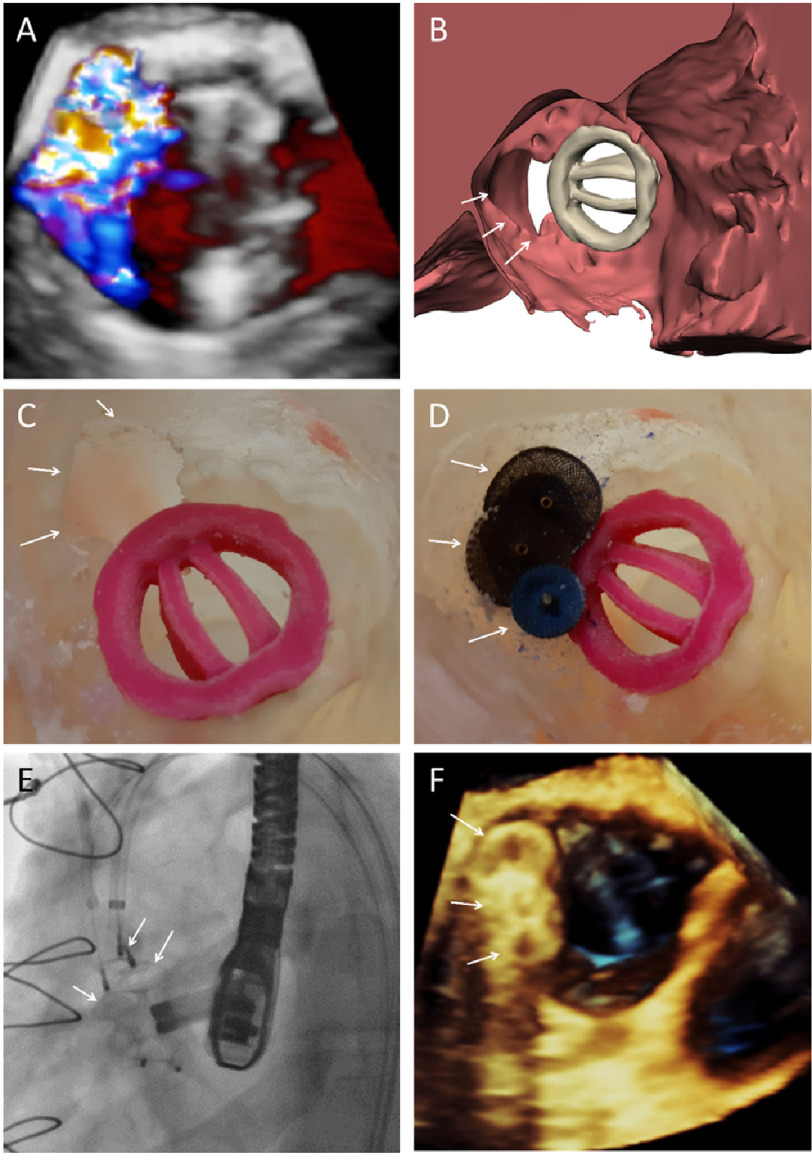
The first case. **A**. 3D TEE showing the defect at 8–11 o’clock. **B.** CT segmentation showing the defect. **C.** 3D model of the defect. **D.** Bench test using three AVP II. **E.** Percutaneous closure using planned plugs. **F.** 3D TEE showing well seated plugs.

A 27-year-old gentleman presented 3 years following his second redo surgical mechanical aortic valve replacement with shortness of breath on mild exertion secondary to severe aortic PVL. The defect was very large measuring (1.8 × 0.6 cm) and extending between 8 and 11 o’clock, as seen by three dimensional transesophageal echocardiography (3D TEE) (Figure 1A). Following a Heart Team meeting, trans-catheter PVL closure was decided.

### Procedural vignette

#### Planning

Segmentation of the computed tomography (CT) images was performed using Materialise Mimics Innovation Suite 21 (Materialise NV, Leuven, Belgium) (Figure 1B). A 3D model was generated and saved as stereo-lithography file format (STL). 3D STL files were then exported to Stratasys polyjet printer Objet260 connex3 (Stratasys, Rehovot, Israel) to be printed using Stratasys Vero family of materials. (Figure 1C). Bench-testing was done using various AVP II devices given concerns about possible interference with the valve occluders (Figure 1D). Eventually we decided to use three Amplatzer vascular plugs (AVP) II devices (sizes 10, 12 and 14 mm) which collectively provided excellent anatomical sealing of the defect without interfering with the occluders of the mechanical prosthesis.

#### Procedure

After obtaining bilateral femoral access, the defect was wired in a retrograde manner using a straight tipped 0.025 inch Terumo guidewire. A 6F AL1 catheter was advanced over the wire which was then exchanged for two Amplatz extra-stiff wires. Two AVP II plugs (12 and 14 mm) were deployed simultaneously through a 9F Cook shuttle delivery sheath followed by another 10 mm plug through a JR 7F guiding catheter (Figure 1E). Post-implantation echocardiography showed trace residual leak with free movement of the occluders (Figure 1F).

## Case Two

### Clinical vignette

**Figure 2.**
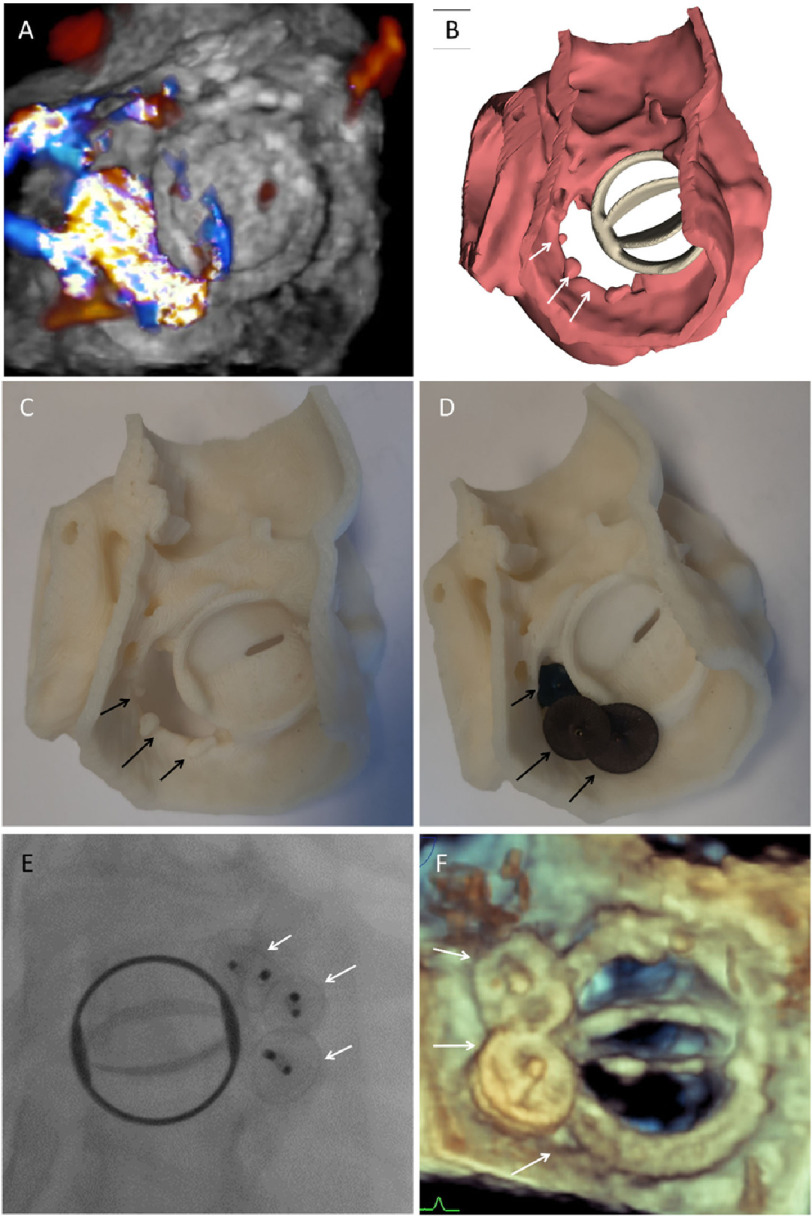
The second case. **A**. 3D TEE showing the defect at 6–10 o’clock. **B.** CT Segmentation showing the defect. **C.** 3D model of the defect. **D.** Bench test using three AVP II. **E.** Percutaneous closure using planned plugs. **F.** 3D TEE showing well seated plugs.

A 50-year-old lady presented with severe mitral PVL 10 years after her second mitral valve replacement surgery. She had refractory hemolytic anemia and NYHA class III heart failure. 3D TEE demonstrated a large crescentic defect, extending between 6 and 10 o’clock and measured (1.7 × 0.9 cm) (Figure 2A).

### Procedural vignette

#### Planning

A CT-derived 3D printed model was performed. Bench-testing was done using various plugs given concerns about possible interference with occluders of the mitral prosthesis. Eventually, we decided to use three AVP II devices (sizes 10, 12 and 12 mm) which provided excellent anatomical sealing of the defect without interfering with the occluders of the mechanical prosthesis. (Figures 2B, 2C, 2D).

#### Procedure

After obtaining transseptal access under TEE guidance, the defect was wired antegradely using a 0.025 inch Terumo wire via a 5F multipurpose catheter aided by an 8.5F deflectable Agilis sheath. The wire was then snared in the ascending aorta and externalized via the contralateral femoral artery forming an arteriovenous loop. Two 12 mm AVP II devices were delivered simultaneously through an 8F Cook shuttle sheath followed by another 10 mm plug via a JR4-6F guiding catheter using anchoring wire technique (Figure 2E). Post-deployment TEE showed trace paravalvular regurgitation (Figure 2F).

## Discussion

Percutaneous closure of paravalvular leaks has emerged as an effective - and often first-line – choice for patients with significant paravalvular regurgitation requiring intervention. The defect can be approached via different routes (antegrade trans-septal, retrograde trans-aortic, trans-apical) based on the position of the prosthesis, location of the defect, number of mechanical prostheses, and operator expertise^3^.

Pre- and intra-procedural evaluation and guidance by 3D echocardiography is critical for preprocedural planning and successful closure^3,4^.

The procedure is sometimes technically challenging due to the location, size and/or shape of the defect as well as the possibility of interfering with the discs of the mechanical valve. This is particularly true in very large defects where the use of more than one large device is anticipated posing both planning and technical challenges^5^.

With very limited existing data on the appropriate sizing algorithms, 3D printed models and preprocedural bench-testing can be extremely useful in such scenarios providing the interventionist with invaluable information on the optimal type, number and size of devices needed.

### Learning points

3D-printed models can be extremely useful for detailed preprocedural planning of percutaneous closure of PVLs, and allow for bench-testing to help identify the optimal type, number, and size of device(s) to be used without encroachment on the valve occluders. This is especially useful in difficult anatomies, very large defects, and in cases with failed previous attempts.

## Disclosure statement

All authors have no conflict of interest to report. Informed consent was obtained from both patients.

## ABBREVIATIONS LIST

AVP: Amplatzer vascular plug
CT: Computed tomography
NYHA: New York Heart Association
PVL: Paravalvular leak
3D TEE: Three-dimensional trans-esophageal echocardiography
